# Macrophage-like cells are still detectable on the retinal surface after posterior vitreous detachment

**DOI:** 10.1038/s41598-022-17229-5

**Published:** 2022-07-27

**Authors:** Jacob M. Wang, Janice X. Ong, Peter L. Nesper, Amani A. Fawzi, Jeremy A. Lavine

**Affiliations:** 1 Department of Ophthalmology, Feinberg School of Medicine, Northwestern University, Chicago, IL USA; 2 Department of Ophthalmology, Feinberg School of Medicine, Northwestern University, 240 E. Huron Street, Bldg. McGaw M343, Chicago, IL 60611 USA

**Keywords:** Monocytes and macrophages, Retina

## Abstract

The identity of vitreoretinal interface macrophage-like cells (MLCs) remains unknown and potential candidates include retinal microglia, perivascular macrophages, monocyte-derived macrophages, and/or vitreal hyalocytes. Since hyalocytes are detectable on the posterior vitreous surface after vitreous extraction in animals, we imaged patients with and without posterior vitreous detachment (PVD) to determine if hyalocytes are the principal MLC component. We performed repeated foveal-centered 3 × 3 mm OCT-A images from 21 eyes (11 no PVD and 10 PVD eyes). Images were registered, segmented, and averaged. The OCT slab from 0 to 3 microns above the internal limiting membrane was used to detect MLCs. We calculated MLC density and distribution in relation to the superficial vascular plexus for 3 vascular regions—on vessels, perivascular, and non-vascular. MLC density was 1.8-fold greater in the PVD group compared to the no PVD group (P = 0.04). MLCs in eyes with PVD were increased 1.9-fold on-vessel (P = 0.07), 1.9-fold in the perivascular region (P = 0.12), and 2.2-fold in non-vascular areas (P = 0.22). MLC density was not severely reduced after PVD, suggesting that the majority of MLCs are not vitreal hyalocytes. PVD status is an important parameter in future MLC studies.

## Introduction

Advanced ocular imaging, including optical coherence tomography^1^ (OCT) and adaptive optics^2^, has identified macrophage-like cells (MLCs) at the vitreoretinal interface in human subjects. On both imaging modalities, these cells were shown to be mobile with a ramified morphology^1,2^, two characteristics that are typical for macrophages. In support of a macrophage identity, *Cx3cr1*^+^ vitreoretinal interface cells have been identified by adaptive optics scanning laser ophthalmoscopy in mice^3^. We recently found that MLCs are increased in patients with proliferative diabetic retinopathy compared to non-proliferative diabetic retinopathy, diabetes without retinopathy, and healthy controls^4^. In addition, another group found increased MLC numbers after retinal vein occlusion^5^. However, the importance of these findings remains unclear since the identity of MLCs remains unknown.

Macrophages are heterogenous cells and possible MLC candidates include microglia, perivascular macrophages, monocyte-derived macrophages, and/or vitreal hyalocytes. Microglia are tissue resident, yolk sac-derived retinal macrophages that primarily exist in the inner and outer plexiform layers. However, microglia populations are also found in the retinal nerve fiber layer that could potentially be seen at the retinal surface^6^. Perivascular macrophages are a distinct cell population from microglia, which have been characterized in the brain^7^ and retina^8^ that are important for maintaining vascular homeostasis and the blood–brain barrier. Since large retinal vessels reside in the retinal nerve fiber layer, perivascular macrophages represent potential MLC candidates. As opposed to steady state microglia and perivascular macrophages, inflammation and breakdown of the blood retinal barrier can stimulate influx of monocyte-derived macrophages into the retina, including to the retinal surface^9,10^. Finally, hyalocytes are resident macrophages of the vitreous body that line the cortical vitreous and stain positively for macrophage markers like CD169, Iba-1, and F4/80^11^. Since MLCs are macrophages detected at the vitreoretinal interface, they may potentially include some or all of these macrophage subtypes.

This study aims to improve our understanding of vitreoretinal interface MLCs by determining if they are principally a part of the retina or vitreous. Posterior vitreous detachment (PVD) is a phenomenon associated with liquefaction of the vitreous gel and separation of the cortical vitreous from the retina. Since prior studies on hyalocytes found that after vitreous extraction, hyalocytes are detected on the vitreous gel surface^12^, we hypothesized that MLCs would be severely reduced after PVD if hyalocytes were the principal MLC component. We used OCT and OCT-angiography (OCT-A) imaging to quantify MLC numbers in patient eyes with and without PVD. We found that MLCs were in fact increased in eyes with PVD, and not severely reduced, suggesting that most MLCs are not vitreal hyalocytes. Furthermore, PVD status is an important potential confounding factor in future MLC studies.

## Results

We included 21 eyes from 21 subjects, including 11 eyes without PVD and 10 eyes with PVD. The demographic characteristics of both groups are shown in Table 1. The two groups were significantly different in age and sex. The PVD cohort was significantly older (age 64.5 ± 7.3 vs 46.4 ± 15.3) and consisted of more females (90% vs 45%) than the healthy controls. There was no significant difference in refractive error or the average Q-score between the two groups.

**Table 1:** Demographic characteristics.

	Groups		*P*
	*No PVD*	*PVD*	
Number of subjects	11	10	–
Age (mean ± SD)	46.4 ± 15.3	64.5 ± 7.3	0.003*
Sex, *n* female (%)	5 (45%)	9 (90%)	0.031*
Refractive error (D mean ± SD)	− 1.16 ± 2.4	− 3.1 ± 3.1	0.144
Missing, *n* (%)	0	2	–
Average Q-score (mean ± SD)	7.6 ± 1.2	7.4 ± 0.7	0.351

*PVD* posterior vitreous detachment, *SD* standard deviation.

*Statistically significant (*P* < 0.05).

**Figure 1:**
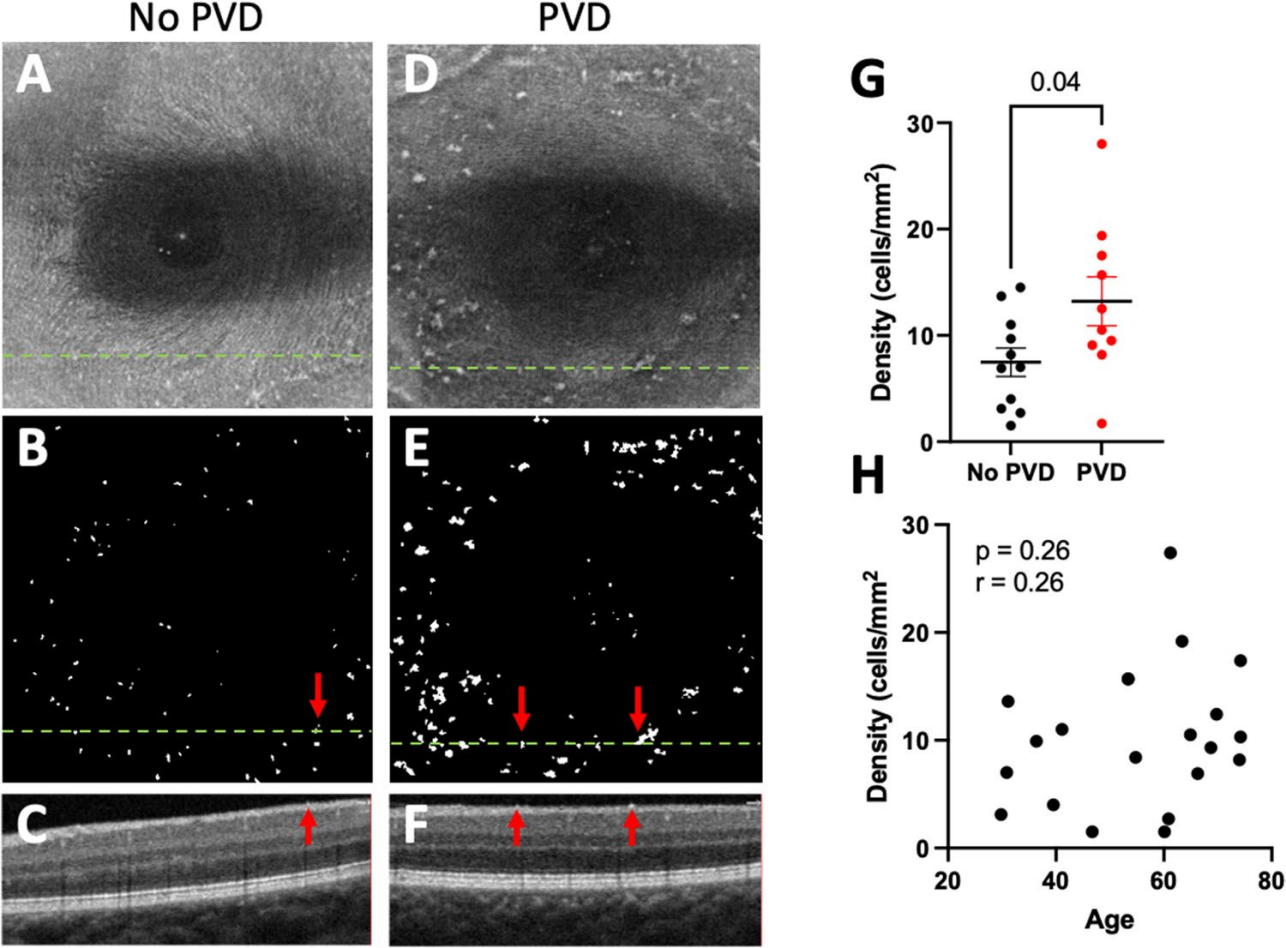
PVD increased MLC density. Representative samples showing MLCs in no PVD (**A**–**C**) and PVD (**D**–**F**) eyes. The aligned and averaged 3 micron OCT slab is displayed on the top row (**A**, **D**); hyperreflective dots represent MLCs. The binarized map of white MLCs is visualized in the second row (**B**, **E**). The green dashed lines denote the location of the B-scan. Representative B-scans are shown on the bottom row (**C**, **F**). Red arrows indicate MLCs identified on the B-scan. MLC density was increased in PVD eyes (*P* = 0.04, **G**). No correlation between MLC density and age was detected (*P* = 0.26, **H**).

Representative images showing MLCs in control (Fig. 1A–C) and PVD eyes (Fig. 1D–F) are shown in Fig. 1A–F. We found that the MLC density was 1.8-fold greater in the PVD group (13.21 ± 7.29 cells/mm^2^) compared to no PVD eyes (7.48 ± 4.43 cells/mm^2^; *P* = 0.04, Fig. 1G). MLC density in our no PVD eyes was very similar to our previously published MLC density^4^ from healthy eyes (6.4 ± 7.0, p = 0.65), highlighting the reproducibility of our methodology. Since our PVD cohort was older, we performed a Pearson’s correlation analysis and found no association between age and MLC density (Fig. 1H). Since MLCs are not severely reduced after PVD, this data suggests that hyalocytes are not the primary MLC cell type.

**Figure 2:**
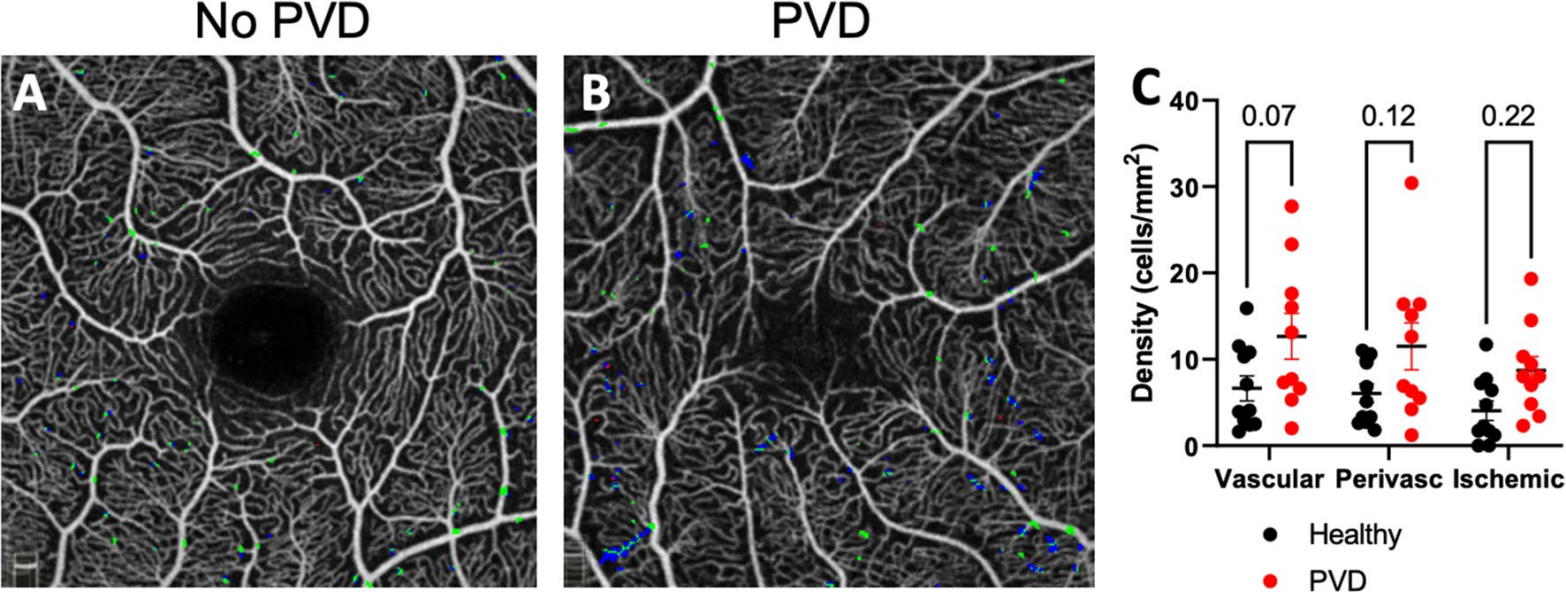
PVD eyes showed a trend toward more MLCs near superficial vessels. Representative images of MLCs in relation to blood vessels in no PVD (**A**) and PVD (**B**) eyes. Green MLCs are on-vessel, blue MLCs are perivascular, and red MLCs are in the ischemic region. MLC density was increased in all three compartments with greater trends in the vascular and perivascular regions compared to the ischemic region (**C**).

Next, we investigated whether increased MLCs are found in a specific location to understand whether increased MLCs are derived from the vasculature. We found that MLCs were increased by 1.9-fold on-vessel (*P* = 0.07), 1.9-fold in the perivascular region (*P* = 0.12), and 2.2-fold in non-vascular areas (*P* = 0.22, Fig. 2).

## Discussion

Since hyalocytes have previously been detected on the vitreous surface in animals after vitreous extraction^12^, we investigated eyes with a PVD to determine if MLCs were severely reduced, which would provide evidence that hyalocytes are the main MLC cell type. We found that MLC density was not decreased in PVD eyes (Fig. 1), suggesting that most MLCs are less likely vitreal hyalocytes and more likely a retinal macrophage cell type. Furthermore, MLC density increased in PVD eyes, suggesting either increased recruitment of MLCs from the circulation or improved detection of pre-existing MLCs following PVD.

Previous studies have shown that vitreoretinal interface MLCs exhibit characteristics typical of macrophages including a ramified morphology^2^, mobility^1^, and *Cx3cr1*^+^ staining in mice^3^. As discussed in the introduction, possible MLC identities include microglia, perivascular macrophages, monocyte-derived macrophages, and/or vitreal hyalocytes. Since MLCs were not reduced or absent after PVD (Fig. 1), our data suggest that many MLCs are not hyalocytes. However, we cannot exclude the possibility that hyalocytes remain at the retinal surface along with remnants of the cortical vitreous after PVD. Histological confirmation in post-mortem eyes would be necessary to examine this question with greater certainty. Based on our data, we hypothesize that MLCs are a mixture of different macrophage sub-types, including microglia and perivascular macrophages at steady state with a minor hyalocyte contribution. This hypothesis is supported by a recent adaptive optics study, which showed that the majority of macrophages on the vitreoretinal interface have a highly ramified morphology and travel short distances, while a minority of cells migrate greater distances with a less ramified morphology^13^. We hypothesize that the less migratory and more ramified vitreoretinal interface macrophages are microglia, while the greater migratory and less ramified cells are hyalocytes.

In addition to greater MLC density in PVD eyes, we found no significant difference in MLC density based upon vascular location (Fig. 2). This finding suggests at least two hypotheses for increased MLC numbers after PVD. First, that MLCs could include monocyte-derived macrophages that extravasate and migrate away from vessels in response to transient injury after PVD. Alternatively, the lack of vitreous could potentially enhance the ability of OCT to detect preexisting microglia and/or perivascular macrophages that were previously masked by the attached vitreous. Further post-mortem and/or animal studies are needed with specific markers for microglia, perivascular macrophages, and monocyte-derived macrophages to better understand the identity and function of MLCs.

A third hypothesis is that PVD-induced MLCs include migrating glial cells. PVD can be an inciting event for epiretinal membrane formation^14^. Muller glia cell migration to the ILM surface and differentiation into myofibroblasts has been proposed as a mechanism for epiretinal membrane pathogenesis^15^. It is therefore possible that increased MLCs after PVD could also include migrating glial cells. Without adaptive optics-OCT confirmation in vivo, which would provide optimal transverse resolution for cell morphology and axial resolution for ILM surface location, or post-mortem histological confirmation, the identity of the increased MLC population after PVD remains uncertain.

Limitations of this study include inherent demographic differences between the cohorts and lack of axial length measurements. The PVD group was significantly older and included more female subjects (Table 1). The effect of sex on MLCs is unknown and requires further investigation. However, increasing age is correlated with reductions, not increases, in MLC density^2^. Our finding of increased MLC density in the older PVD eyes further strengthens our evidence that MLC density is indeed increased by PVD. Furthermore, we found no association between MLC density and age, suggesting that this difference between cohorts did not confound our findings. In addition, most patients included in our study did not have an axial length recorded, which may affect image scaling^16^. However, no difference was found in refractive error, suggesting that axial length is unlikely to confound our data.

In conclusion, our study found that vitreoretinal interface MLC numbers are not significantly reduced after PVD. Our findings suggest that most MLCs are not hyalocytes and are more likely retinal macrophages, including microglia and perivascular macrophages at steady state and potentially monocyte-derived macrophages after injury like during diabetes or retinal vein occlusion. However, we cannot exclude cortical hyalocyte remnants on the retinal surface after PVD or migrating glial cells; further post-mortem and/or animal studies remain necessary to determine the origin, identity, and function of MLCs. Finally, PVD status appears to be an important parameter and potential confounder that needs to be considered in future MLC studies.

## Methods

We conducted a prospective, cross-sectional study of healthy subjects with and without PVD seen between July 2020 and January 2021 in the Department of Ophthalmology at Northwestern University in Chicago, Illinois. The study was approved by the Institutional Review Board of Northwestern University (IRB no. STU00200890) and conducted in accordance with the tenets of the Declaration of Helsinki and regulations of the Health Insurance Portability and Accountability Act. Written informed consent was obtained from all subjects before participation.

Inclusion criteria were patients aged 20–75 years with or without a clinical diagnosis of PVD by a board-certified ophthalmologist and confirmed on OCT imaging. Exclusion criteria were patients with prior eye surgery, retinal disease, vitreoretinal interface pathology like epiretinal membrane or vitreomacular adhesion, or significant media or lens opacity that obscured imaging. In addition, healthy patients with partial PVD detected by OCT were excluded.

### OCTA imaging

Images were obtained using the RTVue-XR Avanti system (Optovue, Fremont, CA, USA) with split-spectrum amplitude-decorrelation angiography (SSADA) software^17^ (version 2017.1.0.151). Repeated foveal-centered 3 × 3 mm (304 × 304 pixels) spectral domain OCT-A images were performed on each patient (mean: 6.0 repeats, range: 4–10). Images with a Q score less than 5 or motion artifact were excluded. For subjects with imaging in both eyes, the eye with higher image quality (average Q score) was included.

### Image analysis

Images were registered using the full retinal vascular network OCT-A slab via the Register Virtual Stack Slices Plugin (Feature Extraction Model = Rigid, Registration Model = Elastic) in FIJI, a distribution of the program ImageJ (National Institutes of Health, Bethesda, MD, USA). Next, the saved transformation matrix was applied to the MLC layer slabs from 0 to 3 µm above the internal limiting membrane^1^ using the Transform Virtual Stack Slices Plugin. Registered MLC stacks were then averaged. MLCs were identified from averaged images using our previously published^4^ semiautomated custom macro in FIJI. Please see our prior publication for a more detailed description, including our high reproducibility of this process (ICC = 0.998, 95% confidence interval = 0.982–0.999, p < 0.05)^4^. We quantified the number of MLCs using the Analyze Particles function, and the MLC density was calculated from the number of MLCs and the total image area (cells/mm^2^).

The location of MLCs in relation to underlying vessels in the superficial vascular plexus was investigated by sorting cells into three vascular regions—on vessels, perivascular (1–30 microns from a vessel), and non-vascular (> 30 microns from the nearest vessel)—using a custom macro in FIJI as previously described^4^. MLCs in the foveal avascular zone (FAZ) were excluded from this analysis because MLCs in the FAZ would be artificially labeled as non-vascular. We excluded the FAZ by removing the entire central 1-mm circle from the analysis. Cell densities were calculated for each region as the number of cells divided by the area of each respective region.

### Statistics

Statistical analysis was performed using GraphPad Prism 9.0.1 (GraphPad Software, San Diego, California USA). We used a two-tailed, unpaired t-test to evaluate the difference between MLC density and demographic data except for age, which was analyzed using Welch’s t-test due to unequal variances between groups. Correlation between age and MLC density was tested with Pearson’s correlation. We used a two-way analysis of variance followed by Šídák's multiple comparisons test to compare MLC densities among the three vascular regions and between the two patient populations.

## Data Availability

The datasets used and analyzed are available from the corresponding author on reasonable request.
